# Risk profile of Qatari women treated for infertility in a tertiary hospital: a case-control study

**DOI:** 10.1186/s40738-020-00080-5

**Published:** 2020-07-27

**Authors:** Sarah Musa, Sherif Osman

**Affiliations:** 1 Department of Family & Community Medicine, Hamad Medical Corporation, Doha, Qatar; 2 Department of Tropical Health, High Institute of Public Health, Alexandria University, Alexandria, Egypt; 3 Department of Family & Community Medicine, Primary Health Care Corporation, Doha, Qatar

**Keywords:** Infertility, Risk factors, Sexual transmitted disease, Modifiable, Prevention

## Abstract

**Background:**

Female infertility is a multifactorial condition constituting a worldwide public health problem. The ability to reproduce is an important product of any marriage, hence infertility may exert a negative impact on physical, financial, social and emotional wellbeing of affected couples. The cornerstone to the management of any disease, including infertility, is prevention. Identifying the modifiable risk factors of female infertility will aid at prevention, early detection, and treatment of medical conditions that can threaten fertility as well as promoting healthy behaviours that can preserve it.

**Aim:**

To explore the risk profile of infertility among Qatari women and compare risk factors distribution among primary vs. secondary infertility.

**Methodology:**

A hospital-based case control study was conducted from September 17th, 2017- February 10th, 2018. Cases (*n* = 136) were enrolled from infertility clinic and controls (pregnant women, *n* = 272), were enrolled from antenatal clinic, Women Hospital, Hamad Medical Corporation (HMC). Interview questionnaire was utilized to collect data about sociodemographic, risk factors related to infertility and patient health Questionnaire (PHQ)-2. Body Mass Index (BMI) was calculated. Logistic regression was used to identify the associated factors to infertility. Statistical significance was set at 0.05.

**Results:**

Forty three primary and ninety three secondary infertility cases were included. Risk factors were age >  35 years (OR = 3.7, 95% CI: 1.41–9.83), second-hand smoking (OR = 2.44, 95% CI:1.26–4.73), steady weight gain (OR = 4.65,, 95% CI: 2.43–8.91), recent weight gain (OR = 4.87, 95% CI: 2.54–9.32), menstrual cycle irregularities (OR = 4.20, 95% CI:1.14–15.49), fallopian tube blockage (OR = 5.45, 95% CI: 1.75–16.95), and symptoms suggestive of sexually transmitted infections (STIs) including chronic lower abdominal/pelvic pain (OR = 3.46, 95% CI: 1.57–7.63), abnormal vaginal discharge (OR = 3.32, 95% CI:1.22–9.03) and dyspareunia (OR = 7.04, 95% CI: 2.76–17.95). Predictive factors for secondary infertility were; longer time from previous conception (OR = 5.8, 95% CI: 3.28–10.21), history of stillbirth (OR = 2.63, 95% CI: 1.04–6.67) or miscarriage (OR = 2.11, 95% CI: 1.21–3.68) and postpartum infection (OR = 3.75, 95% CI: 1.27–11.06). Protective factors were higher education level (OR = 0.44, 95% CI: 0.25–0.78), higher income (OR = 0.17, 95% CI: 0.06–0.49), and awareness/loyalty to fertility window (OR = 0.33, 95% CI: 0.21–0.52 and OR = 0.29, 95% CI: 0.19–0.44, consequently).

**Conclusion:**

This study highlighted the opportunities to strengthen public health as well as hospital-based health promotion programs importantly toward behavioural-related risk factors (e.g. smoking, obesity, STIs etc.). Moreover, detecting, preventing, and managing modifiable risk factors through awareness, screening and early management of chronic diseases, may contribute at reduction of incidence and severity of infertility. Such interventions can be delivered at premarital, family planning, post-natal and antenatal clinics at primary health care with early referral to secondary care if required.

## Plain English summary

Infertility is defined by the failure to conceive after 1 year or more of regular unprotected sexual intercourse. It is considered as a stigmatizing condition more pronounced in Arab communities. Couples are distracted by the physical, financial, social and emotional hardship of the disease. It can also affects marriage stability, family relationships and job performance. Although male and female are attributed equally to infertility (third of cases each), it appears that women is consistently held responsible and she is often impacted psychologically and socially as a consequence. Several risk factors of female infertility might be preventable particularly the ones related to behaviour and lifestyle.

This study attempts to explore the risk factors of female infertility to provide guidance for prevention and early management. We have interviewed infertile females (136) and fertile pregnant females (272) using questionnaires individually. We have classified infertility as primary (women with no previous conception) or secondary (women with previous conception).

Of the 136 infertile cases, 43 had primary infertility and 93 had secondary infertility. We found that the most associated risk factors to female infertility were age >  35 year, second hand smoking, steady weight gain since marriage, recent weight gain, irregular menstrual cycle, fallopian tube blockage, some symptoms that can be related to sexual transmitted infections including chronic lower abdominal pain, abnormal vaginal discharge, and pain during sexual intercourse. Risk factors for secondary infertility were identified as the following; history of stillbirth/miscarriage, postpartum infection or previous caesarean section. Higher education/income as well as awareness/loyalty to fertility window, were found to be protective against infertility.

In conclusion, infertility is a multifactorial disease that remain a significant burden for individuals, families and communities. Several modifiable risk factors were found to be associated with female infertility, which may be considered for planning of better reproductive healthcare in Qatar.

## Key message points

- Lifestyle pattern mainly obesity and second-hand smoking, is contributed to the occurrence of female infertility among Qatari women.
- Screening for symptoms suggestive of sexual transmitted disease is an essential step for prevention of female infertility.
- Secondary female infertility is found to be linked to the rate of caesarean section, stillbirth and miscarriages.

## Introduction

Infertility is a disease of the reproductive system defined by the failure to achieve a clinical pregnancy after 12 months or more of regular unprotected sexual intercourse [1, 2]. Primary infertility is defined as the inability to conceive after 1 year of unprotected sexual intercourse, with no previous conceptions, while secondary infertility is referred to couples who are unable to conceive after 1 year of unprotected intercourse following a previous pregnancy [3, 4]. About one-third or more of all infertility cases are related to women’s causes, another third due to male causes, the remaining are caused by mixed or by unknown factors [5]. Globally, every year, 60–80 million new couples suffer from infertility [6]. A systematic analysis published by the World Health Organization (WHO) in 2012, revealed that one in every four couples in developing countries are affected with infertility [2]. Infertility affects between 8 and 12% of reproductive-aged couples worldwide [6, 7]. However, in some regions, the rates are much higher, reaching up to 30% in some populations such as Middle East and North Africa (MENA) region [7–9]. Infertility is a cause of instability in the lives of couples, particularly women, raising chances of divorce, lowering chances of entering into marriage, put her at risk of family violence, and increasing the chances that her husband will marry another wife, in religions where polygyny is permitted, as in the Islamic Arab world [10]. Treatment of infertility can be medically invasive, associated with adverse health problems and my cause psychological stress, anxiety or depression. A serious risk of ovulation induction is ovarian hyperstimulation syndrome (OHSS). Some published research suggests that infertility treatments may be associated with an increased risk of gynaecologic or breast cancer. Infertility treatments have increased the rate of twin and higher-order multiple births, which put both mother and infants at higher risk of adverse health outcomes. Even singleton births resulting from Assisted Reproductive Technology (ART) are associated with increased risk of low birth weight (LBW) and even at higher risk of birth defects. Lack of access to public health care, traditional means of self-cure (e.g. unprotected sex with multiple partners to achieve the goal of a wanted pregnancy) can result in the spread of HIV and other STIs, with the potential to contribute further to the disease burden [11]. Female infertility risk factors ranges from non-modifiable such as older age, ethnic background, congenital anomalies of reproductive organ, certain genetic conditions, family history [12–14], and modifiable factors that include sociodemographic, STIs, post-abortal or postpartum infections leading to fallopian tube blockage, high risk sexual behaviour (e.g. early age at first sexual intercourse, multiple marriages/relations), environmental hazards (e.g. radiation exposure, chemotherapeutic and toxic agents), lifestyle factors (e.g. obesity, tobacco smoking, alcohol intake, emotional stress, etc.), some medical conditions (as menstrual cycle abnormalities, thyroid diseases, polycystic ovarian syndrome (PCOS)), and prior history of pelvic surgeries (e.g. caesarean section, appendectomy) [15–18]. According to the United Nation’s (UN) “World Population Prospects”: The 2015 Revision; total fertility rate in Qatar has dropped from 6.11 children per woman in 1965–1980 to 2.1 in 2010–2015. Projections show that total fertility will decline further to reach 1.76 in 2020–2025, which is below the replacement level fertility. The most important factors they unveiled are as increased age at first marriage, increased educational level of Qatari women, and more women integrated in the labour force [19]. The aim of the present study was to explore infertility risk profile among Qatari females that will aid in planning preventive and management strategies to mitigate its burden, and consequently maternal and foetal morbidity, mortality and economic cost on families and on the healthcare system.

## Method section

### Study design

An analytical case-control study was conducted.

### Study settings and duration

Cases were recruited from infertility clinics, Women Hospital-HMC, Doha. It is the main governmental hospital providing infertility counselling and management services in the State of Qatar, where most cases are served on the national level coverage. The clinic serves around 3500 patients annually, at an average rate of 300 patients per month. For the year 2017, the clinics covered 1486 new cases as well as 1973 follow up cases. Among those, 42% were Qatari women. Controls were recruited from the antenatal clinic, Women Hospital-HMC, Doha. Antenatal clinics at Women Hospital are the main provider of such service within secondary care level in Qatar, parallel to Primary Health Care Corporation (PHCC). The clinic serves around 60,000 patients annually, at an average rate of 5000 patients per month. For the year 2017, the clinics covered 10,657 new cases and 48,503 follow-up cases. Among those, 40% were Qatari women. The study was conducted during the period from 17th September 2017 to 10th February 2018.

### Target population

#### Inclusion criteria

##### Cases

Defined as; any Qatari women within the reproductive age (15–49 years), who reports failure to achieve a clinical pregnancy after 12 months or more of regular unprotected sexual intercourse, attending infertility clinic at Women’s Hospital - HMC. **Controls:** Defined as; any Qatari pregnant woman within the reproductive age (15–49 years), attending antenatal clinic at Women Hospital - HMC. Controls are supposed to be those seeking healthcare (antenatal care) at the same setting (Women’s Hospital) and mostly attributed to the same population pool where cases came from. A ratio of 2:1 was utilized for controls to cases.

#### Exclusion criteria

##### For cases

Those with clinical diagnosis of infertility due to male or combined causes.

##### For controls

Those with prior complain/history of infertility or previously managed to treat infertility and those with the current pregnancy being a product of infertility management.

### Sample size calculation and sampling technique

Sample size of 408 (136 cases and 272 controls) was calculated using the following case-control study formula [20]:

***n*** = $$ \frac{\left(r+1\right)}{r}X\frac{\left(\ P\ \right)\ x\ \left(\ 1-P\ \right)\ x\ \left(\  Z\beta + Z\alpha\ \right)2}{\left(P1-P2\right)2.} $$

Where:

***n*:** Minimum sample size required [for the cases group]

***r*:** Ratio of control to cases [i.e. 2: 1] = 2

***Z***_***α***_**:** Standard normal variant for the selected significance level [i.e. 95%] = 1.96

***Z***_***β***_**:** Standard normal variant for the desired 80% power = 0.84

***OR*:** The assumed least *Odds Ratio* foreseen = 2

***P:*** Average proportion exposed

***P***_***1***_**:** The assumed proportion exposed in the case group that is calculated as the following:

$$ P\; cases\;\mathit{\exp}o=\frac{OR\times P\; controls\kern0.17em expo}{p\; controls\kern0.17em expo\;\left( OR-1+1\right)} $$

***P***_***2***_**:** The assumed proportion exposed in the control group, where three different proposed risk factors of infertility were reviewed in literature to acquire their prevalence in the studied community. They were the following; *Qatari women suffering chlamydial infection (5.3)* [21], *polycystic ovarian syndrome (18.33%)* [22] *and, obesity (36.4%)* [23]

The average for the three was calculated to be 20%.

### Sampling technique

Cases were recruited using a convenient non-probability sampling technique. Controls were selected from those pregnant women attending the antenatal clinic, using probability systematic random sampling technique. List of attendees at the daily appointment sheet was used as a sampling frame where participants were selected systematically each fourth listed, after selecting the first one randomly. The average Qatari women attending the clinic /month = 2000. The clinic runs AM/PM shifts 5 days a week. Average daily attendance AM shift = 50 (two stations each 25 cases/station/shift). The sampling interval (k) was calculated based on the following formula [***k = N/n***], where **N** is the population size = 2000/2 shits = 1000 divided by ***n*** = 272 = 3.67 rounded into 4.

### Research instruments

Data were collected using predesigned interview questionnaire consisting of the following components; Sociodemographic characteristics (age, education level, occupation, and income), marriage history (consanguinity, age at first marriage, recurrent marriage, duration of marriage, husband’s absence), lifestyle history (smoking, alcohol, vigorous exercise, weight gain), menstrual history (age of menarche, regularity of menstrual cycle, duration of menstrual cycle, number of menstrual flow days, menorrhagia, intermenstrual bleeding, dysmenorrhea, secondary amenorrhoea, obstetric history (previous and time of previous conception, stillbirth, miscarriage, ectopic pregnancy, antenatal care, post-partum/abortal infection, gynaecologic history (chronic pelvic pain, abnormal vaginal discharge, painful urination, dyspareunia, gynaecological related fever, pelvic inflammatory disease (PVD), tubal blockage, fibroid uterus, endometriosis or congenital anomaly of the reproductive organ, medical history (diabetes mellitus (DM), thyroid disease, hyperprolactinemia), medication history (cancer treatment, prolonged use of steroid, hormonal therapy, prolonged high dose of nonsteroidal anti-inflammatory drugs (NSAIDs), certain antihypertensive, anti-obesity, antidepressant/antipsychotic), surgical history (caesarean section, dilatation & curettage, appendectomy, pelvic or abdominal surgery), birth control history (contraception use and methods; oral contraceptive pills, intrauterine device, natural/barrier methods), family history (female infertility, menstrual cycle irregularity, early menopause, PCOS, fibroid uterus, DM, thyroid disease), sexual history (knowledge & loyalty to fertility window, coital frequency). The second component of the questionnaire was PHQ – 2 and any patients who scored positive were advised to get referral into a specialized care for further evaluation with the more explicit and specific PHQ-9. Medical review was performed as well as anthropometric measurement of weight, height and body mass index (BMI).

### Study variables

#### Dependent (outcome)

##### Primary infertility

Women in the reproductive age group who are unable to conceive after 1 year of unprotected sexual intercourse with no previous conceptions.

##### Secondary infertility

Women in the reproductive age group who are unable to conceive after 1 year of unprotected intercourse following a previous pregnancy.

##### Independent

Included sociodemographic characteristics, history of marriage, lifestyle, menstrual, obstetric, gynaecological, medical, medication, surgical, birth-control, sexual and family, depression screening using patient health questionnaire (PHQ)-2 score, and anthropometric measurements. BMI was calculated and classified according to the World Health Organization (WHO).

### Ethical considerations

Formal approvals were obtained prior to field work from the Arab Board of Medical Specialization, Research Ethics Committee of Women Hospital, Medical Research Center (MRC)-HMC and Institutional Review Board (IRB)-HMC. Informed consent was taken from the willing participants after explaining the aim, objectives and possible benefits from the study following the HMC-IRB standard template of informed consent. All eligible clients were participating totally voluntarily and given the chance to clarify any concerns. The study was conducted with no negative effect on the relationship between the clients and the healthcare provider. Clients were instructed that they could withdraw at any time without any adverse consequences. Confidentiality of the information and privacy have been assured throughout the study. Those screened as positive by the PHQ-2 were advised to go further with the PHQ-9 testing at specialized secondary care.

### Quality control measures

Content and face validity of the constructed questionnaire were established by extensive literature review, consultation of experts in the fields of community medicine, maternal health, primary health care and consultants in obstetrics and gynaecology specializing in infertility. The principle investigator performed data collection with the assistance of an assigned data collector (physician). Adequate training of the data collector was done through explaining in details all sections of the questionnaire, as well as, interviewing few clients in front of the assigned physician. The researcher reviewed the questionnaires to ensure completion and consistency, together with extracting the pre-conception weight from electronic medical records to calculate BMI. Prior to data collection, the questionnaire was piloted using a convenient sample of 10 eligible cases and 10 controls to test for the clarity, understandability, feasibility and timeliness to complete the questionnaire. Those piloted participants were later omitted. The completed questionnaires were reviewed on daily basis and revised for data completion and consistency by the PI.

### Data analysis

Data entry was done using Statistical Package of Social Science *IBM-SPSS*© version 22. *Student t-test* and *chi square* test were used to compare (mean + standard deviation) and (observed frequency) for numerical and categorical variables, consequently. Crude and adjusted odd ratios (OR) were calculated to examine the risk association between two variables. Variables having *p*-value equal or less than 0.05 at the bi-variable analysis were considered as statistically significant and were further included in the multivariate logistic regression. Two regression model using forward stepwise method were used; *Model I* was to obtain risk factors of primary and secondary infertility compared to controls, while *Model II* was to obtain risk factors of secondary infertility compared to controls with previous conception.

## Patient and public involvmement

Patients were involved in identifying research priorities. They were interviewed during rotations at infertility clinic to identify the most important and relevant outcome measures. Patients worked with us in formulating the research questions, however it was difficult to involve patients in other areas of the study design due to data protection restriction and ethical considerations. Dissemination strategies will include raising awareness of preventive risk factors of female infertility among Qatari through media such as television programmes, newspaper and social media. Moreover, leaflets will be designed for Primary Health Care Centers to be available at premarital clinics, post-natal clinic and well-women clinic, as well as infertility clinics related to Hamad Medical Corporation.

## Results

**Fig. 1:**
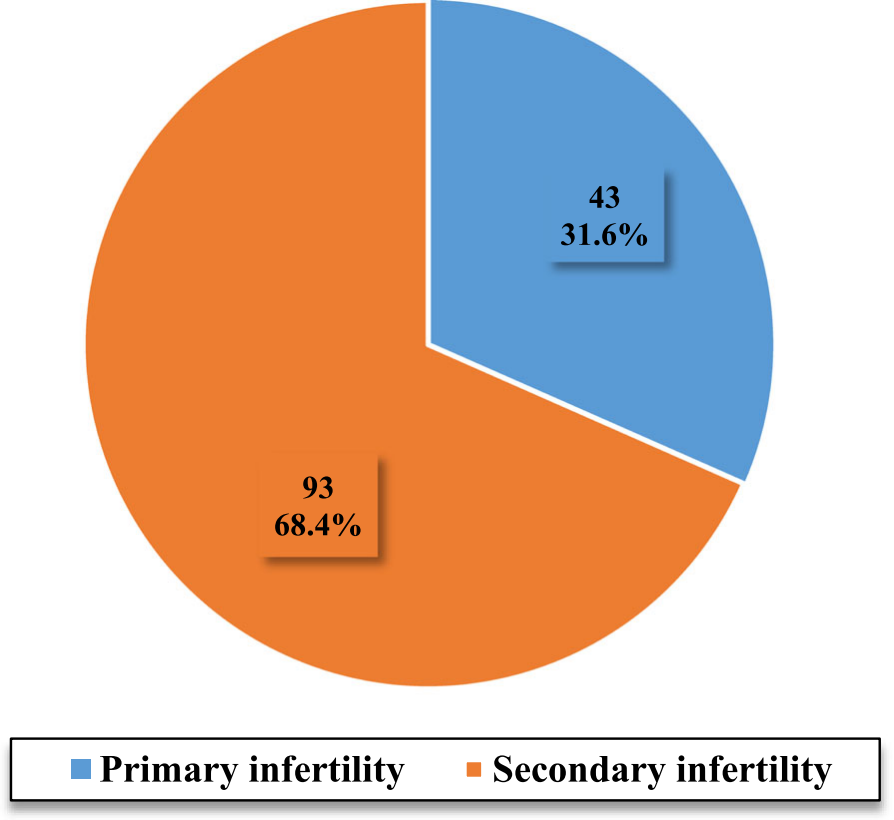
Distributions of infertile participants according to fertility type, Women Hospital-Hamad Medical Corporation, 2018

It was found that 68.4% of infertile participants were suffering from secondary infertility, while the remainder (36.6%) had primary infertility. Fig. 1.

Table 1 shows the distribution of cases and controls according to their sociodemographic characteristics. The mean age of cases and controls was 32.5 + 6.6 years and 30.2 + 5.5 years, subsequently. Regarding the educational level, majority of participants in both groups have completed secondary and/or university education or higher. More than half of cases and more than three quarter of controls had their average monthly income in the high category (> 25.000 Qatari Riyals). Occupation showed no statistical difference between the two groups. Regarding the age at first marriage, 11.7% of infertile women got married at an age of 30 year or above as compared to only 5.1% of controls (*p* = 0.024). In respect to husband’s absence, only 14.7% of control reported their husbands being absent from home, compared to as high as 31.6% of infertile participants, the difference was statistically significant (*p* = 0.001). However, consanguinity, recurrent marriage and duration of menstrual cycle had no statistical significance between groups.

**Table 1:** Distribution of study participants according to their socio-demographic characteristics, Women Hospital-Hamad Medical Corporation, 2018

Sociodemographic	Cases		Cases	Controls	***p*** value
	Primary Infertility ***n*** = 43 (%)	Secondary Infertility ***n*** = 93 (%)	Infertility Total n = 136 (%)	n = 272(%)	
**Age group:**					0.002*
16–25 year	12 (27.9)	12 (12.9)	24 (17.7)	59 (21.7)	
26–35 year	23 (53.5)	43 (46.2)	66 (48.5)	164(60.3)	
> 35 year	8 (18.6)	38 (40.9)	46 (33.8)	49 (18.0)	
**Mean** **+** **SD**	**29.4** **+** **6.7**	**33.9** **+** **6.0**	**32.5** **+** **6.6**	**30.2** **+** **5.5**	
**Educational level:**					0.028*
Illiterate	1 (2.3)	5 (5.4)	6 (4.4)	2 (0.7)	
Primary	6 (14.0)	4 (4.3)	10 (7.4)	13 (4.8)	
Preparatory	1 (2.3)	11 (11.8)	12 (8.8)	13 (4.8)	
Secondary	17 (39.5)	40 (43.0)	57 (41.9)	131(48.2)	
University / higher	18 (41.9)	33 (35.5)	51 (37.5)	113(41.5)	
**Average household monthly income:**					
< 12.000 QR	5 (11.6)	6 (6.5)	11 (8.1)	5 (1.8)	0.001*
12.000- < 25.000 QR	13 (30.2)	33 (35.5)	46 (33.8)	52 (19.1)	
> 25.000 QR	25 (58.1)	54 (58.1)	79 (58.1)	215(79.0)	
**Age at first marriage:**					0.024*
< 20 years	6(14.0)	26(28.0)	32(23.5)	86(31.6)	
20 - < 25	18(41.9)	37(39.8)	55(40.4)	113(41.5)	
25 - < 30	10(23.3)	23(24.7)	33(24.3)	59(21.7)	
30 - < 35	4(9.3)	5(5.4)	9(6.6)	12(4.4)	
> 35	5(11.6)	2(2.2)	7(5.1)	2(0.7)	
**Mean** **+** **SD**	**25.8** **+** **6.8**	**22.7** **+** **4.6**	**23.7** **+** **5.6**	**22.1** **+** **4.4**	
**Husband’s absence:**					0.001*
*Never been absent*	33 (76.7)	60 (64.5)	93 (68.4)	232 (85.3)	
Yes, occasionally	4 (9.3)	17 (18.3)	21 (15.4)	19 (7.0)	
Yes, frequently/seasonally	4 (9.3)	12 (12.9)	16 (11.8)	16 (5.9)	
Yes, most of the time	2 (4.7)	4 (4.3)	6 (4.4)	5 (1.8)	
*Yes, “collectively”*	10(23.3)	33 (35.5)	43 (31.6)	40 (14.7)	

* *p* < 0.05

*SD* Standard Deviation, *QR* Qatari Riyals

Table 2 shows the distribution of study participants according to their lifestyle history. Only 2.2% of cases are currently cigarette smokers, compared to none of their fertile counterparts, who reported never being smokers either currently or previously. Similarly, nine cases (6.6%) are currently or previously smoked water pipe tobacco, while only 1.5% of controls have similar exposure, the difference was statistically significant (*p* = 0.006). Around 58.1% of cases reported exposure to second hand smoke, the figure was significantly higher than their controls (*p* = 0.014). None of the study participants reported alcohol consumption. Infertile participants reported practicing vigorous exercise (as swimming, fixed cycling and jugging) more commonly that their controls, 8.8 and 3.3% respectively (*p* = 0.017). Around one fourth of cases had history of childhood obesity, while the majority of them reported steady weight gain since the start of marriage and/or recently during the last 6 months. On the other hand, controls significantly showed much lower figures.

**Table 2:** Distribution of study participants according to their lifestyle-related characteristics, Women Hospital-Hamad Medical Corporation, 2018

Lifestyle history	Cases		Cases	Controls	***p*** value
	Primary Infertility n = 43 (%)	Secondary Infertility n = 93 (%)	Infertility Total n = 136 (%)	n = 272 (%)	
**Water pipe smoking:**					0.005*
No	41 (95.3)	86 (92.5)	127 (93.4)	268 (98.5)	
Yes, currently	1 (2.3)	5 (5.4)	6 (4.4)	3 (1.1)	
Yes, previously	1 (2.3)	2 (2.2)	3 (2.2)	1 (0.4)	
Yes, *“collectively”*	2 (4.7)	7 (7.5)	9 (6.6)	4 (1.5)	
**Second hand smoking:**					0.006*
No	15 (35.9)	42 (45.2)	57 (42.0)	153 (56.3)	
Yes, currently	17 (39.5)	41 (44.1)	58 (42.6)	103 (37.9)	
Yes, previously	11 (25.6)	10 (10.8)	21 (15.4)	16 (5.9)	
Yes, *“collectively”*	28 (65.1)	51(54.8)	79 (58.1)	119 (43.8)	
**Vigorous exercise:**					0.017*
No	40 (93.0)	84 (90.3)	124 (91.2)	263 (96.7)	
Yes	3 (7.0)	9 (9.7)	12 (8.8)	9 (3.3)	
**Childhood obesity:**					0.004*
No	34 (79.1)	70 (75.3)	104 (76.5)	238 (87.5)	
Yes	9 (20.9)	23 (24.7)	32 (23.5)	34 (12.5)	
**Steady weight gain since marriage:**					0.001*
No	11 (25.6)	25 (26.9)	36 (26.5)	203 (74.6)	
Yes	32 (74.4)	68 (73.1)	100 (73.5)	69 (25.4)	
**Recent weight gain:**					0.001*
No	15 (34.9)	38 (40.9)	53 (39.0)	232 (85.3)	
Yes	28 (65.1)	55 (59.1)	83 (61.0)	40 (14.7)	

* *p* < 0.05

Table 3 demonstrates the distribution of study participants according to gynaecological history. Majority of the cases and controls had normal age of menarche. Cases were more likely to report history of mensural cycle irregularity of duration more than 6 months, as well as history of menorrhagia, intermenstrual bleeding, dysmenorrhea and secondary amenorrhea, with statistical significance differences. Symptoms suggestive of STIs (chronic pelvic pain, abnormal vaginal discharge, painful urination, dyspareunia) were highly significant among cases as compared to controls. Gynaecological related-fever had no statistical significance difference.

**Table 3:** Distribution of study participants according to gynaecological history, Women Hospital-Hamad Medical Corporation, 2018

Gynecological history	Cases		Cases	Controls	***P*** value
	Primary Infertility n = 43 (%)	Secondary Infertility ***n*** = 93 (%)	Infertility Total n = 136 (%)	n = 272 (%)	
**Menstrual cycle regularity:**					0.001*
Regular	32 (74.4)	65 (69.9)	97 (71.3)	256 (94.1)	
Irregular	11 (25.6)	28 (30.1)	39 (28.7)	16 (5.9)	
**Menstrual irregularity:**	***n*** **= 11**	***n*** **= 28**	***n*** **= 39**	***n*** **= 16**	0.001*
< 6 month	1 (9.1)	4 (14.3)	5 (12.8)	8 (50.0)	
≥ 6 month	10 (90.9)	24 (85.7)	34 (87.2)	8 (50.0)	
**Duration of menstrual cycle:**					0.001*
< 21 days	0 (0.0)	1 (1.1)	1 (0.7)	0 (0.0)	
21–35 days	33 (76.7)	61 (65.6)	94 (69.1)	252 (92.6)	
> 35 days	10 (23.3)	31 (33.3)	41 (30.2)	20 (7.4)	
**Menorrhagia:**					0.001*
No	31 (72.1)	72 (77.4)	103 (75.7)	260 (95.6)	
Yes	12 (27.9)	21 (22.6)	33 (24.3)	12 (4.4)	
**Intermenstrual bleeding:**					0.001*
No	38 (88.4)	84 (90.3)	122 (89.7)	272(100.0)	
Yes	5 (11.6)	9 (9.7)	14 (10.3)	0 (0.0)	
**Dysmenorrhoea:**					0.001*
No	30 (69.8)	74 (79.6)	104 (76.5)	256 (94.1)	
Yes	13 (30.2)	19 (20.4)	32 (23.5)	16 (5.9)	
**Secondary amenorrhea:**					0.001*
No	31 (72.1)	67 (72.0)	98 (72.1)	258 (94.9)	
Yes	12 (27.9)	26 (28.0)	38 (27.9)	14 (5.1)	
**Chronic pelvic pain**					0.001*
No	27 (62.8)	52 (55.9)	79 (58.1)	255 (93.8)	
Yes	16 (37.2)	41 (44.1)	57 (41.9)	17 (6.2)	
**Abnormal vaginal discharge:**					0.001*
No	36 (83.7)	64 (68.8)	100 (73.5)	261 (96.0)	
Yes	7 (16.3)	29 (31.2)	36 (26.5)	11(4.0)	
**Painful urination:**					0.001*
No	38 (88.4)	80 (86.0)	118 (86.8)	269 (98.9)	
Yes	5(11.6)	13(14.0)	18 (13.2)	3 (1.1)	
**Dyspareunia:**					0.001*
No	25 (58.1)	55 (59.1)	80 (58.8)	262 (96.3)	
Yes	18 (41.9)	38 (40.9)	56 (41.2)	10 (3.7)	

* *p* < 0.05

Table 4 shows the distribution of secondary infertility participants and controls according to their obstetric history**.** Most of secondary infertile cases and controls had their previous pregnancy within last 5 years. Secondary infertile women were more likely to report history of stillbirth, recurrent miscarriage, post-partum/abortal infection, caesarean section, while history of ectopic pregnancy or dilatation & curettage were not found to be statistically significant. Around 15% of secondary infertile cases reported not having antenatal care in their previous pregnancies, compared to only 7.8% of controls, the difference reached statistical significance.

**Table 4:** Distribution of secondary infertility participants and controls according to their obstetric history, Women Hospital-Hamad Medical Corporation, 2018

Obstetric history	Secondary infertility n = 93 (%)	Controls n = 272 (%)	***p*** value
**Previous conception:**			0.001*
No	0 (0.0)	42 (15.4)	
Yes	93 (100.0)	230 (84.6)	
**Time from previous conception:**	**n = 93**	***n*** **= 230**	0.001*
< 5 years	47 (50.5)	194 (84.3)	
5 - < 10 years	38 (40.9)	32 (14.0)	
10 - < 15 years	4 (4.3)	4 (1.7)	
≥ 15 years	4 (4.3)	0 (0.0)	
**Outcome of previous conception:** - **Stillbirth**		**n = 230**	0.004*
Never	80 (86.0)	219 (95.2)	
Happened once or more	13 (14.0)	11 (4.8)	
**Miscarriage**			0.021*
Never	35 (37.6)	124 (53.9)	
Once	35 (37.6)	57 (24.8)	
Twice or more	23 (24.8)	49 (21.3)	
**Post-partum / post-abortal infection:**		**n = 230**	0.005*
No	83 (89.2)	223 (97.0)	
Yes	10 (10.8)	7 (3.0)	
**Caesarean section:**			0.042*
No	55 (59.1)	192 (70.6)	
Yes	38 (40.9)	80 (29.4)	

* *p* < 0.05

Table 5 demonstrates the distribution of study participants according to medical/medication history. Hypothyroidism, hyperprolactinemia, depression were reported significantly higher among cases. More than half of the infertility cases were suffering from PCOS, versus 19.1% of their controls. Furthermore, around 17% of cases had fallopian tube blockage, compared to only 2.6% of their fertile controls. Secondary infertile women tended to have higher rate of fallopian tube blockage than women with primary infertility (20.4% vs. 9.3% respectively). Fibroid uterus was reported among 19.6% of cases compared to only 4.0% of controls. Endometrioses and reproductive congenital anomalies showed no statistical significance. More cases reported history of appendectomy compared to controls (8.3% vs. 3.3% respectively). Furthermore, the rate of surgical management of obesity (most commonly sleeve gastrectomy and/or liposuction) was significantly higher among cases compared to their controls (24.3% vs. 13.6% respectively). History of other pelvic surgeries was statistically more frequent amongst cases than controls (18.4% vs. 15.1% respectively). Cases were more likely to have history of prolonged use of steroid, hormonal therapy, prolonged high dose of NSAID, and anti-obesity. However, cancer treatment, anti-hypertensive and antidepressant showed no statistical significant difference.

**Table 5:** Distribution of study participants according medical/medication history, Women Hospital-Hamad Medical Corporation, 2018

Medical/ Medication history	Cases		Cases	Controls	***p*** value
	Primary Infertility n = 43 (%)	Secondary Infertility n = 93 (%)	Infertility Total n = 136 (%)	n = 272 (%)	
**Hypothyroidism:**					0.025*
No	35 (81.4)	75 (80.6)	110 (80.9)	242 (89.0)	
Yes	8 (18.6)	18 (19.4)	26 (19.1)	30 (11.0)	
**Hyperprolactinemia:**					0.001*
No	34 (79.1)	78 (83.9)	112 (82.4)	255 (93.8)	
Yes	9 (20.9)	15 (16.1)	24 (17.6)	17 (6.2)	
**Depression and/or other psychological disorders:**					0.002*
No	40 (93.0)	88 (94.6)	128 (94.1)	270 (99.3)	
Yes	3 (7.0)	5 (5.4)	8 (5.9)	2 (0.7)	
**Polycystic ovarian syndrome:**					0.001*
No	21 (48.8)	43 (46.2)	64 (47.1)	220 (80.9)	
Yes	22 (51.2)	50 (53.8)	72 (52.9)	52 (19.1)	
**Fibroid uterus:**					0.026*
No	37 (86.0)	86 (92.5)	123 (90.4)	261 (96.0)	
Yes	6 (14.0)	7 (7.5)	13 (19.6)	11 (4.0)	
**Appendectomy**					0.035*
No	41 (95.3)	84 (94.3)	125 (91.9)	263 (96.7)	
Yes	2 (4.7)	9 (9.7)	11 (8.3)	9 (3.3)	
**Prolonged steroid:**					0.050*
No	42 (97.7)	84 (90.3)	126(92.6)	267 (98.2)	
Yes	1 (2.3)	9 (9.7)	10 (7.4)	5 (1.8)	
**Hormonal therapy:**					0.001*
No	15 (34.9)	19 (20.4)	34 (25.0)	260 (95.6)	
Yes	28 (65.1)	74 (79.6)	102(75.0)	12 (4.4)	
**Prolonged high dose of NSAID:**					0.001*
No	40 (93.0)	78 (83.9)	118(86.8)	261 (96.0)	
Yes	3 (7.0)	15 (16.1)	18 (13.2)	11 (4.0)	
**Anti-obesity** **(*Xenical*®,*Meridia*®):**					0.001*
No	42 (97.7)	81 (87.1)	123(90.4)	267 (98.2)	
Yes	1 (2.3)	12 (12.9)	13 (9.6)	5 (1.8)	

* *p* < 0.05

*NSAID* Non-Steroidal Anti Inflammatory Drugs

*Xenical*® is the trade name for Orlistat, *Meridia*® is the trade name for Sibutranine

Table 6 shows the distribution of study participants according to their birth-control/sexual history. Among contraception users, hormonal control was the most commonly adopted method (71.7 and 50% among cases and controls subsequently), followed by natural/barrier method. However, the use of intrauterine devices as well as duration of birth control use, showed no statistical significant difference between the two groups. Controls were more likely to be aware and loyal to fertility window, while coital frequency showed no statistical significance difference.

**Table 6:** Distribution of study participants according medical/medication history, Women Hospital-Hamad Medical Corporation, 2018

	Cases		Cases	Controls	***p*** value
	Primary Infertility	Secondary Infertility	Infertility Total		
**Birth -control/ sexual history**	**n = 43 (%)**	**n = 93 (%)**	**n = 136 (%)**	**n = 272 (%)**	
**Previous postpone of child bearing:**					0.001*
No	38 (88.4)	45 (48.4)	83 (61.0)	88 (32.4)	
Yes	5 (11.6)	48 (51.6)	53 (39.0)	184 (67.6)	
**Hormonal control:**	***n*** **= 5**	***n*** **= 48**	***n*** **= 53**	***n*** **= 184**	0.006*
No	2 (40.0)	13 (27.1)	15 (28.3)	92 (50.0)	
Yes	3 (60.0)	35 (72.9)	38 (71.7)	92 (50.0)	
**Birth control through natural / barrier methods:**	**n = 5**	***n*** **= 48**	**n = 53**	**n = 184**	0.004*
No	3 (60.0)	39 (81.2)	42 (79.2)	106 (57.6)	
Yes	2 (40.0)	9 (18.8)	11 (20.8)	78 (42.4)	
**Awareness about fertility window:**					0.001*
No	18 (41.9)	38 (40.9)	56 (41.2)	51 (18.8)	
Yes	25 (58.1)	55 (59.1)	80 (58.8)	221 (81.2)	
**Loyalty to fertility window:**					0.001*
No	24 (55.8)	51 (54.8)	75 (55.1)	71 (26.1)	
Yes	19 (44.2)	42 (45.2)	61 (44.9)	201 (73.9)	

* *p* < 0.05

**Table 7:** Distribution of study participants according to their family history, Women Hospital-Hamad Medical Corporation, 2018

Family history	Cases		Cases	Controls	***p*** value
	Primary Infertility n = 43 (%)	Secondary Infertility n = 93 (%)	Infertility Total n = 136 (%)	n = 272 (%)	
**Female infertility:**					0.004*
No	24 (55.8)	55 (59.1)	79 (58.1)	197 (72.4)	
Yes	19 (44.2)	38 (40.9)	57 (41.9)	75 (27.6)	
**Menstrual cycle irregularity:**					0.001*
No	28 (65.1)	63 (67.7)	91 (66.9)	227 (83.5)	
Yes	15 (34.9)	30 (32.3)	45 (33.1)	45 (16.5)	
**Polycystic ovarian syndrome:**					0.050*
No	30 (69.8)	69 (74.2)	99 (72.8)	221 (81.3)	
Yes	13 (30.2)	24 (25.8)	37 (27.2)	51 (18.8)	
**Fibroid:**					0.004*
No	34 (79.1)	75 (80.6)	109 (80.1)	246 (90.4)	
Yes	9 (20.9)	18 (19.4)	27 (19.9)	26 (9.6)	
**Diabetes mellitus:**					0.001*
No	16 (37.2)	35 (37.6)	51 (37.5)	197 (72.4)	
Yes	27 (62.8)	58 (62.4)	85 (62.5)	75 (27.6)	
**Thyroid disease:**					0.001*
No	30 (69.8)	55 (59.1)	85 (62.5)	219 (80.5)	
Yes	13 (30.2)	38 (40.9)	51 (37.5)	53 (19.5)	

* *p* < 0.05

Table 7 demonstrates the distribution of study participants according to their family history. Family history of female infertility was observed in 41.9% of cases compared to 27.6% of controls. Moreover, family history of menstrual cycle irregularity, PCOS, fibroid, DM and thyroid disease were all more distributed among cases, with a significant statistical difference.

Table 8 illustrates the distribution of study participants according to their WHO-BMI classification/ PHQ-2 results. The mean BMI values for cases was higher compared to their controls (mean + standard deviation = 31.4 + 6.4 Kg/m^2^ and 28.7 + 6.1 Kg/m^2^, respectively Infertile women were more likely to be obese as compared to controls with statistical significance difference. With regard PHQ-2 results, 14% of infertile women screened positive against depression compared to only 5.5% of their controls, with a statistical significant difference.

**Table 8:** Distribution of study participants according to their WHO-BMI classification/ PHQ-2 score, Women Hospital-Hamad Medical Corporation, 2018

WHO-BMI classification	Cases		Cases	Controls	***p*** value
	Primary Infertility n = 43 (%)	Secondary Infertility n = 93 (%)	Infertility Total n = 136 (%)	n = 272 (%)	
**Underweight**	0 (0.0)	0 (0.0)	0 (0.0)	7 (2.6)	0.002*
**Normal weight**	6 (13.9)	15 (16.1)	21 (15.4)	68 (25.0)	
**Overweight**	14 (32.6)	27 (29.0)	41 (30.2)	98 (36.0)	
**Obese**	23 (53.5)	51 (54.9)	74 (54.4)	99 (36.4)	
**Mean BMI** **+** **SD**	**30.9** **+** **6.2**	**31.6** **+** **6.6**	**31.4** **+** **6.4**	**28.7** **+** **6.1**	0.001*
**PHQ-2 score result:**					0.004*
Negative	38 (88.4)	79 (84.9)	117 (86.0)	257	
Positive	5 (11.6)	14 (15.1)	19 (14.0)	(94.5) 15 (5.5)	

* *p* < 0.05

**Fig. 2:**
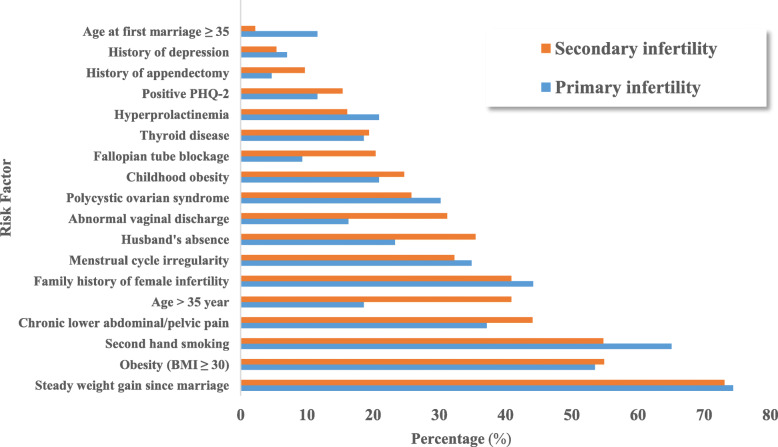
Distribution of some risk factors among primary and secondary infertile participants, Women Hospital-Hamad Medical Corporation, 2018

**Fig. 3:**
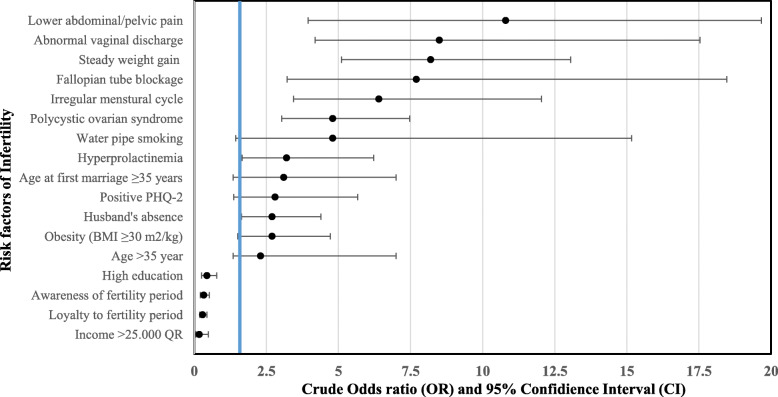
Main significant risk factors of infertility among Qatari women derived from the bivariate analysis, Women Hospital-Hamad Medical Corporattion, 2018

Comparing the distribution of selected significant risk factors between primary and secondary infertility in bivariate analysis, it was found that husband’s absence, older age, abnormal vaginal discharge, fallopian tube blockage, history of appendectomy and older age at first marriage, were more commonly found among secondary infertile women. Fig. 2. The most predictive factors of infertility obtained after bivariate analysis, illustrated with crude OR and 95% confidence interval (CI) are shown in Fig. 3.

Table 9 describes the result of multivariate logistic regression analysis. Among the forty two entered significant factors only nine were found to be predictors of infertility [*Model I: X*^2^ (12) = 264, *p* < 0.001] including; age > 35 years, second hand smoking, steady weight since marriage, recent weight gain, menstrual cycle irregularity, chronic lower abdominal pain, abnormal vaginal discharge, dyspareunia and fallopian tube blockage. Furthermore, four variables were found to be predictors of secondary infertility (among those with history of previous conception) [*Model II: X*^2^ (4) = 57.3, *p* < 0.001], including duration of 5 years or more from previous conception, stillbirth, recurrent miscarriage and post-partum/abortal infection.

**Table 9:** Infertility risk factors: Results of the bivariate and multivariate logistic regression analysis, Women Hospital-Hamad Medical Corporation, 2018

Independent risk factors	Crude Odd ratio (cOR)	95% Confidence interval (CI)	Adjusted Odd ratio (aOR)	***p-***value	95% Confidence interval (CI)
**Model I: Independent risk factors of infertility**^a^					
**Age of participants (> 35 y)**	2.31	1.24–4.29	3.72	0.008*	1.41–9.83
**Second hand smoking**	1.78	1.18–2.70	2.44	0.008*	1.26–4.73
**Steady weight gain since marriage**	8.17	5.11–13.05	4.65	0.001*	2.43–8.91
**Recent weight gain**	9.08	5.62–14.69	4.87	0.001*	2.54–9.32
**Menstrual cycle irregularity**	6.43	3.44–12.04	4.20	0.031*	1.14–15.49
**Sexually transmitted infections (STIs) suggestive symptoms:**					
Lower abdominal/pelvic pain	10.82	3.95–19.67	3.46	0.002*	1.57–7.63
Abnormal vaginal discharge	8.54	4.19–17.44	3.32	0.019*	1.22–9.03
Dyspareunia	18.44	8.95–37.60	7.04	0.001*	2.76–17.95
**Fallopian tube blockage**	7.71	3.22–18.47	5.45	0.003*	1.75–16.95
**Model II: Independent risk factors of secondary infertility**^b^					
**Time from last conception ≥ 5y**	4.9	2.78–8.65	5.8	0.001*	3.28–10.21
**History of stillbirth (once or more)**	3.24	1.39–7.52	2.63	0.042*	1.04–6.67
**Miscarriage (once or more)**	1.94	1.18–3.17	2.11	0.008*	1.21–3.68
**Post-partum / Post-abortal infection**	3.84	1.42–10.42	3.75	0.016*	1.27–11.06

* *p* < 0.05

^a^ Model I: X^2^ (12) = 264, *p* < 0.001, *Nagelkerke R*^*2*^ = 0.661

^b^ Model II: X^2^ (4) = 57.3, *p* < 0.001, *Nagelkerke R*^*2*^ = 0.233

Model I included comparison between total cases (n = 136) and total controls (n = 272)

Model II included comparison between secondary infertile women (n = 93) and only controls who reported previous conception (n = 230)

## Discussion

The studied sample revealed that 68.4% had secondary infertility, while 31.6% had primary infertility. Similarly, a systematic analysis [2] of national health surveys conducted among 190 countries by Mascarenhas MN et al. (2012) found that secondary infertility was more prevalent than primary (10.5% vs. 1.9% respectively). However, regionally, a study in Kuwait [24] conducted by Omu FE and Omu AE (2002–2007), revealed that among 268 women attending infertility clinic, the rate of primary and secondary infertility were 65.7 and 34.3%, respectively. As compared to our study, the variation in the distribution of primary and secondary infertility could be related to the selection of different population and exclusion of cases with male factor, where many primary infertility exists.

### Female infertility risk factors

In the current study, It was found that age > 35 years significantly increased the risk of infertility by around four times, (OR = 3.72, 95% CI: 1.41–9.83, *p* = 0.008). It also revealed that with increasing age, the trend of infertility risk increases in a step manner. This is in consonance with a case-control study conducted in Lusaka, Zambia by Kalima-Munalula MN et al. (2017) who found a significant association between age and female infertility. There was an increasing trend of infertility risk, with increasing age, at age group 20 - 29y, the OR was 2.39; and OR of 8.42 at 30 - 39y [25]. Decreased fecundity with increasing female age has long been recognized from demographic and epidemiological studies, which consistently found that fertility declined beginning as early as the middle of the third decade. The biological basis of this decline include decline in the number of oocytes from birth to menopause, the quality of existing oocytes diminishes with age and on an average, intercourse frequency declines with age [26].

This study found that second hand smoking (aOR = 2.44, 95% CI = 1.26–4.73, *p* = 0.008) and water-pipe smoking were significantly associated with female infertility (OR = 4.75, 95% CI = 1.44–15.71, *p* = 0.01). In agreement to our study, the association between second-hand smoking and infertility was assessed in a prospective cohort of postmenopausal women by Hyland A. et al. (1993–1998). The study established that active-smokers were 1.14 times more likely to have infertility and 1.26 times more likely for earlier menopause than never-smoking women [27]. Second hand smoking was linked to early menopause in several studies that may contribute to female infertility. Moreover, the present study showed that obesity is a significant risk factors for female infertility. This was consistent with the results of a case-control study of 582 women, Algeria by MAÏ HA et al. (2015). It reported that women with BMI greater than 30 m^2^/kg were 3.26 times more likely to have infertility (OR = 3.26) [28]. Similarly, a study conducted in Saudi Arabia, King Fahad Medical City by Rafique M. et al. (2016), revealed that among 127 cases of female infertility, 33.2% were overweight and 48% were obese. In addition, PCOS was present in 30.8% of overweight and 38.7% of obese women [29]. This is not surprising, because obesity is associated with anovulation, menstrual disorders, miscarriage, and adverse pregnancy outcomes, all of which could contribute to the infertile status.

In the current study, it was found that menstrual cycle irregularity is a significant risk of female infertility (aOR = 4.20, 95% CI = 1.14–15.49, *p* = 0.031) including oligomenorrhea, menorrhagia, dysmenorrhea and intermenstrual bleeding. Similarly, Shamila S et al. (2011) in their survey found that menstrual cycle irregularity was a common observation reported among infertile females in the three study areas (40, 44.85 and 44.11% respectively) and was positively correlated with female infertility [30]. Likewise, a case-control study in south-eastern Iran conducted by Ansari H et al. (2016), reported that women with irregular menstruation were nearly 4 times more likely to have secondary infertility, compared to their regular cycle counterparts (aOR = 3.91) [31]. A study conducted in Korea by Kwon SK et al. (2014), found that among the studied 1080 women suffering secondary amenorrhea, PCOS was the most common cause (48.4%) [32]. It was also found in this study that PCOS increased the risk of female infertility by nearly 5 times and these results correlate with the studies concerning the percentage of women suffering infertility problems due to PCOS by Wendy A et al. (54.6%) [33], Susan M. et al. (40%) [34] and Kristi P et al. (56%) [35]. In PCOS, levels of hormones including androgens and testosterone increase due to high levels of luteinizing hormone (LH) and low levels of the follicular-stimulating hormone (FSH), so follicles in these individuals are prevented from producing a mature egg. Furthermore, PCOS increases the risk of insulin resistance, along with type 2 diabetes, which is one of the causes of infertility [36].

The present study found that symptoms suggestive of STIs were highly correlated with female infertility; dyspareunia (OR = 7.04, 95% CI = 2.76–17.95, *p* = 0.001), while chronic lower abdominal pain or abnormal vaginal discharge increased the risk of infertility by more than three times. In Nigeria, Ogbu GI*.* et al. (2017) studied the relationship between *Chlamydia trachomatis* infection and tubal infertility found a statistically significant association between positive *C. trachomatis* antibody titre among cases with tubal factor infertility (75.0%) compared with controls (22.2%). They concluded that the clinical feature having the potential of identifying woman at high risk for *Chlamydia* infection were vaginal discharge (24.5%), followed by dysmenorrhea (24.5%) and lower abdominal pain (23.1%) [37]. The present study also demonstrated that fallopian tube blockage is a risk factor for female infertility (OR = 5.45, 95% CI = 1.75–16.95, *p* = 0.003). Fallopian tube blockage was much more common in secondary infertile females (20.4%) compared to only 9.3% of primary infertile. Tubal blockage is usually associated with chronic untreated STIs/PID or could be related to history of adverse pregnancy outcome, both of which, calls for the urgency of implementing STIs screening program and appropriate antenatal and post-natal care consequently.

In this study, hypothyroidism and hyperprolactinemia were found to be predictors for female infertility. This was also seen in a study by Hymavathi k et al. (2016), India to investigate the correlation of thyroid and prolactin hormones levels with female infertility. The study found that 27% of women with primary infertility were hypothyroid and 7% were hyperthyroid. Among those with secondary infertility the corresponding figures were 5 and 2% respectively. Additionally, hyperprolactinemia was detected in 37% of infertile cases, more commonly among primary infertile women (79.4%) as compared to 20.6% secondary infertile [38]. Thyroid dysfunction have been found to be associated with anovulatory cycles, decreased fecundity, and increased morbidity during pregnancy. Hyperprolactinemia also adversely affects the fertility potential by disturbing pulsatile secretion of GnRH and hence interfering with ovulation. It may result in menstrual and ovulatory dysfunctions like anovulation, amenorrhea and galactorrhoea. In addition, history of appendectomy was found to be an independent risk factor for female infertility, in present study. On contrary, a meta-analysis by Elraiyah T et al. (2014) showed that previous appendectomy is not significantly associated with increased incidence of infertility in women, (OR = 1.03) [39]. Complicated, ruptured appendicitis has been implicated in causing scarring, which can lead to infertility and/or ectopic pregnancy.

Awareness and loyalty to fertility window were found in the current study to be protective against infertility. A cross-sectional study of fertility-awareness among women seeking fertility assistance in Australia by Hampton KD et al. (2013) found that 68.2% believed they had timed intercourse mainly within the fertile, but only 12.7% could accurately identify this window. Most infertile women were graded by the study as having either no fertility-awareness (11.8%) or poor fertility-awareness (52.5%) [40]. Additionally, another study by Blake D et al. (1997), has investigated the fertility-awareness of infertile women seeking fertility assistance, they found that 74% of participants could not accurately identify the fertile window [41]. There is a compelling need to educate women about their fertility awareness. Primary care providers need to integrate fertility health literacy into health promotion of women of reproductive age.

### Secondary infertility risk factor

The current study revealed that history of recurrent miscarriages/stillbirth was as twice as common among female with secondary infertility. History of post-partum / post-abortal infection and caesarean section were also found to be significant predictors of secondary infertility in this study. In agreement, Dhont N et al. (2009) in their study conducted in Rwanda, found that secondary infertile women were two times more likely to have history of an adverse pregnancy outcome (miscarriage / ectopic pregnancy, (aOR =1.89), history of stillbirth (aOR = 7.52), history of postpartum infection (aOR = 11.49) or history of caesarean section (aOR = 11.49) compared to their controls [42]. The decision for caesarean intervention should not be taken lightly and should be clinically justified.

### Study strengths

This is the first study in Qatar to explore the risk factors of female infertility among Qatari women. Controls were selected from the same population where cases came from, and screening for male factors was done using semen analysis, both of which would minimize selection bias. Since this is unmatched case-control study, multiple logistic regression was applied to overcome the effect of confounders. A ratio of 2:1 (controls to cases) was utilized to increase the statistical power of the study.

### Study limitations

The findings of this study should be considered with the following limitations. First, this is a hospital-based study and findings may not be representative for the general population. Furthermore, controls were selected by probability systematic random sampling technique, while cases were selected via convenient non-probability technique. As a result, the study can be subjected to selection bias which affect the generalizability and the statistical significance of the results. Due to the retrospective nature of case-control studies, recall bias could increase the likelihood that infertile women recall and report exposures compared to their controls, pregnant women. Moreover, temporal relationships between studied risk factors and female infertility cannot be ascertained.

## Conclusion

Infertility is a multifactorial complex disease that remains a significant burden for the individuals, families and communities. Several modifiable risk factors were found to be predictors of female infertility among Qatari females that maybe be considered for planning of better reproductive health care. Older age and delayed age at first marriage beyond 30 years were found to be independent risk factors for infertility. Lifestyle pattern including smoking whether water pipe of second hand, obesity, as well as symptoms suggestive of STIs can contribute significantly to infertile status. Furthermore, menstrual cycle abnormalities, PCOS, tubal blockage, fibroid, hyperthyroidism, hyperprolactinemia, appendectomy, post-partum infection, caesarean section, recurrent miscarriage, stillbirth, were all found to be risk factors of female infertility. Conversely, higher education/income and fertility window awareness were found to be protective against infertility. Therefore, primary prevention as well as screening and early management using cost-effective interventions targeting mainly modifiable risk factors are essential components of reproductive health care planning. Moreover, delivering integrated care through utilization of premarital, well women, antenatal, postnatal, and family planning clinics to raise awareness and screen for related risk factors.

## Data Availability

The data that support the findings of this study are available from Hamad Medical Corporation (HMC) but restrictions apply to the availability of these data, which were used under license for the current study, and so are not publicly available. Data are however available from the authors upon reasonable request and with permission of Medical Research Center-HMC.

## Abbreviations

HMC: Hamad Medical Corporation
PHCC: Primary Health Care Corporation
PHQ-2: Patient health questionnaire
STIs: Sexually transmitted infections
ART: Assisted Reproductive Technology
LBW: Low birth weigh
WHO: World Health Organization
MENA: Middle East and North Africa
PCOS: Polycystic ovarian syndrome
LH: Luteinizing hormone
FSH: Follicular-stimulating hormone
UN: United Nation
PVD: Pelvic inflammatory disease
DM: Diabetes mellitus
NSAIDs: Nonsteroidal anti-inflammatory drugs
BMI: Body mass index
IBM-SPSS: Statistical Package of Social Science
